# FFT-RDNet: A Time–Frequency-Domain-Based Intrusion Detection Model for IoT Security

**DOI:** 10.3390/s25154584

**Published:** 2025-07-24

**Authors:** Bingjie Xiang, Renguang Zheng, Kunsan Zhang, Chaopeng Li, Jiachun Zheng

**Affiliations:** 1 School of Ocean Informattion Engineering, Jimei University, Xiamen 361000, China; jerryxiang@jmu.edu.cn (B.X.); jchzheng@jmu.edu.cn (J.Z.); 2 State Grid Fujian Electric Power Co., Ltd., Zhangzhou Power Supply Company, No. 13 Shengli East Road, Xiangcheng District, Zhangzhou 363000, China; zhengrenguang1983@gmail.com (R.Z.); zhangkunsan1991@gmail.com (K.Z.)

**Keywords:** internet of things, FFT, depthwise separable convolution, residual network, hybrid sampling

## Abstract

Resource-constrained Internet of Things (IoT) devices demand efficient and robust intrusion detection systems (IDSs) to counter evolving cyber threats. The traditional IDS models, however, struggle with high computational complexity and inadequate feature extraction, limiting their accuracy and generalizability in IoT environments. To address this, we propose FFT-RDNet, a lightweight IDS framework leveraging depthwise separable convolution and frequency-domain feature fusion. An ADASYN-Tomek Links hybrid strategy first addresses class imbalances. The core innovation of FFT-RDNet lies in its novel two-dimensional spatial feature modeling approach, realized through a dedicated dual-path feature embedding module. One branch extracts discriminative statistical features in the time domain, while the other branch transforms the data into the frequency domain via Fast Fourier Transform (FFT) to capture the essential energy distribution characteristics. These time–frequency domain features are fused to construct a two-dimensional feature space, which is then processed by a streamlined residual network using depthwise separable convolution. This network effectively captures complex periodic attack patterns with minimal computational overhead. Comprehensive evaluation on the NSL-KDD and CIC-IDS2018 datasets shows that FFT-RDNet outperforms state-of-the-art neural network IDSs across accuracy, precision, recall, and F1 score (improvements: 0.22–1%). Crucially, it achieves superior accuracy with a significantly reduced computational complexity, demonstrating high efficiency for resource-constrained IoT security deployments.

## 1. Introduction

In modern society, characterized by the rapid advancement of the Internet and information technologies, interconnected digital infrastructures—particularly Internet of Things (IoT) ecosystems—have permeated critical industries and daily life. This hyper-connectivity generates massive data exchanges but also facilitates increasingly sophisticated cyber-attacks targeting vulnerable IoT endpoints and distributed infrastructures. Escalating cyber security threats include compromised network endpoints [1], malicious data injections into sensor networks [2], and privacy breaches across distributed systems [3]. These evolving threats, ranging from legacy protocol exploitation to coordinated intrusions, pose severe risks to industrial control systems, smart infrastructure, and national security, challenging the security of distributed architectures and data integrity.

Intrusion detection systems (IDSs) [4] provide critical safeguards by monitoring network traffic for malicious patterns. In IoT ecosystems with resource-constrained endpoints and heterogeneous data, traditional IDSs face significant challenges. These systems are broadly classified as Network IDSs (NIDSs) [5] for monitoring inter-device communications and Host-Based IDSs (HIDSs) [6] for securing embedded devices. Modern NIDSs must address evolving threats like DDoS attacks [7], malicious firmware updates [8], and endpoint spoofing [9], requiring capabilities such as inspecting encrypted traffic and adapting to dynamic IoT topologies [10] while maintaining energy efficiency.

**Figure 1:**
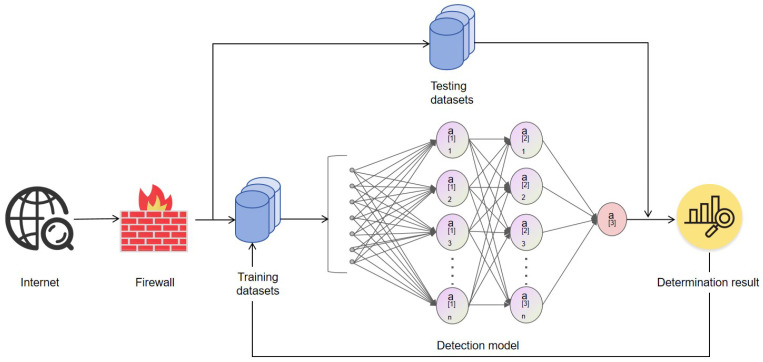
Component diagram of intrusion detection system.

A flowchart of a traditional IDS is illustrated in Figure 1. Initially, network traffic passes through the system firewall, after which point the detection system extracts and segments it. The segmented traffic data is used to train the intrusion detection model, which leverages both historical event data and the characteristics of the current traffic. Subsequently, live test traffic is evaluated by the model to determine whether it represents normal behavior. The determination outcome is then fed back into the training dataset for continuous learning and improvement. If the system identifies abnormal traffic, it triggers an alert and initiates a response according to predefined protocols.

Traditional machine-learning-based IDS algorithms (e.g., decision trees [11] and support vector machines [12]) increasingly struggle with the high dimensionality and complexity of modern network data, particularly from distributed IoT nodes [13,14]. Their limitations in handling spatio-temporal patterns and extracting deep semantic features from encrypted traffic hinder generalization across dynamic environments. Consequently, deep-learning-based IDS approaches have emerged as superior solutions for processing multivariate IoT data streams [15], leveraging strengths in feature extraction, their representational learning capability, and generalization. State-of-the-art architectures include lightweight CNNs [16], RNNs/LSTMs [17,18] for temporal analysis, GANs [19], and GNNs [20] for modeling system interdependencies.

Despite their advantages, these advanced deep learning frameworks face significant drawbacks under the stringent constraints of IoT environments. Primarily, complex CNN and RNN variants impose substantial computational and memory overheads, hindering their deployment on low-power IoT devices—a limitation exacerbated in GNNs due to inherent graph processing demands. Furthermore, while capable, the existing architectures often fail to optimally extract the most discriminative and computationally efficient features from raw network traffic, particularly lacking the ability to concurrently capture localized temporal patterns and broader frequency-domain characteristics. Additionally, models like standard CNNs and RNNs are sensitive to the severe class imbalance prevalent in intrusion datasets, frequently necessitating auxiliary techniques that increase the complexity. Beyond these issues, GAN-based approaches, despite their promise for synthetic data generation, often suffer from training instability and mode collapse, thereby reducing their reliability for robust real-time detection.

Addressing these challenges necessitates the development of a lightweight yet highly effective IDS framework tailored to the IoT. Such a framework must simultaneously deliver a high detection accuracy, low computational complexity, resilience to data imbalances, and efficient multi-modal feature extraction. To this end, we propose FFT-RDNet, a comprehensive network intrusion detection system spanning data preprocessing to model deployment. Our approach utilizes advanced sampling techniques to mitigate data imbalances. Crucially, we propose a novel intrusion detection model operating within a dual-path architecture, concurrently processing features in the time and frequency domains in parallel. This design enables the detection of abnormal patterns through analyzing altered frequency distributions, capturing both transient high-frequency events (e.g., burst attacks [21]) and persistent low-frequency anomalies (e.g., stealthy data exfiltration). Our key contributions are summarized as follows:

(1) Inspired by the frequency characteristics of different network attacks, we model the features of network attacks from the time and frequency domains, respectively. By converting one-dimensional features into two-dimensional features, the extraction of the network traffic’s features is improved.
(2) We propose a network intrusion detection model FFT-RDNet based on a time–frequency-domain analysis. Through depthwise separable convolution and residual networks, the two-dimensional variations between different features of different attacks are captured from the transformed two-dimensional features.
(3) Experiments were conducted on the NSL-KDD dataset and the CIC-IDS2018 dataset. These experiments demonstrate that the proposed method outperforms most of the existing model structures in multiple indicators. The ablation experiments verify the effectiveness of the different modules of the system.

## 2. Related Work

Early intrusion detection methods were largely based on rule matching [22] and feature comparison [23], relying on static analyses of homogeneous network traffic. However, these approaches proved unsuitable for modern IoT ecosystems with heterogeneous devices and encrypted traffic, particularly for detecting zero-day attacks [24] against IoT protocols or complex variants in smart infrastructures.

Deep learning has significantly advanced network intrusion detection, offering enhanced generalization through automated feature extraction. However, its application to the IoT faces challenges due to extreme data imbalances [25] due to diverse device behaviors and the resource constraints of IoT deployments. To address data imbalances, sampling techniques like oversampling, undersampling [26], SMOTE [27], cluster-based undersampling [28], and ADASYN [29] remain predominant strategies.

The most critical challenge in IoT intrusion detection is automatic feature extraction from heterogeneous device network traffic, whereas the traditional techniques rely on manual feature engineering unsuitable for encrypted IoT payloads. Vinayakumar et al. [30] proposed a CNN-based model extracting spatial features to improve the detection accuracy. However, a CNN’s local-feature focus limits its ability to capture remote dependencies in device-to-gateway communication sequences. Recurrent neural networks address this by modeling the temporal dependencies across IoT behavioral logs. Laghrissi et al. [31] proposed an LSTM-based model identifying traffic attack time dependencies, achieving an excellent performance on multiple datasets. In 2019, Liu et al. [32] proposed an FFT-based intrusion detection method converting traffic into frequency-domain images, transforming detection into image classification while ignoring the original time-domain features. In 2020, Sinha et al. [33] proposed a CNN-BiLSTM framework using bidirectional sequence information, reducing the computational overhead significantly, particularly valuable for resource-constrained IoT deployments. In 2021, Sun et al. [34] combined multi-head attention with BiLSTM to identify complex attacks, dynamically weighting the features to detect IoT-specific anomalies like protocol handshake failures. In 2024, Farhan et al. [35] proposed a Transformer+CNN+LSTM model extracting semantic features from encrypted IoT traffic via transformer transfer learning, with LSTM-CNN performing a deep attack analysis. In 2024, Sana et al. [36] applied the Vision transformer to the field of anomaly detection for the first time, expanding the application of ViT models beyond image classification. However, the computational cost of the ViT model is very high, especially in real-time traffic monitoring applications.

Building on this work, we observe that most feature extraction operates in one-dimensional space. We propose a model combining a time–frequency analysis [37], residual networks [38], and depthwise separable convolution (DSC) [39]. This transfers IoT traffic features to two-dimensional space, enabling parallel weighting of the time and frequency features. Residual networks capture the temporal dependencies, while depthwise separable convolution reduces the computational complexity—crucial for real-time IoT security. A comparison between previous methods and the proposed model is presented in Table 1.

## 3. The Proposed Model

### 3.1. The Overall Framework

**Figure 2:**
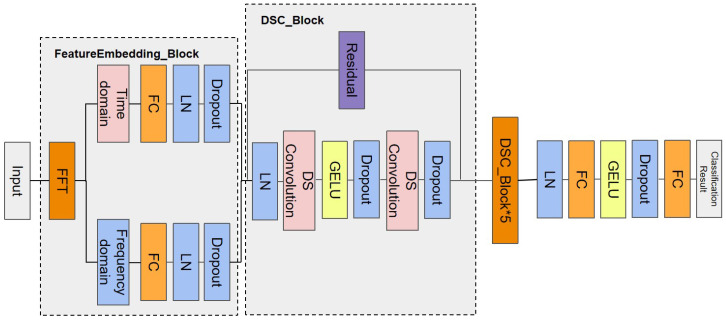
The structural flowchart of FFT-RDNet.

The architecture of FFT-RDNet is illustrated in the flowchart in Figure 2. First, one-dimensional (1D) network traffic features are transformed into a two-dimensional time–frequency representation through the feature embedding module. This module processes the time-domain and frequency-domain features separately before fusing them, enabling the model to efficiently capture the multi-dimensional characteristics of the input data. The embedded features undergo Layer Normalization (LN) and are then processed by a six-layer stack of identical basic blocks. Each basic block comprises a depthwise separable convolution layer, the GELU activation function [41], and regularization components. The local features are extracted via depthwise separable convolution, while residual connections link all structures to progressively integrate multi-scale features from local to global contexts. Finally, the model generates network traffic predictions through a classification head, which outputs category probabilities via a two-layer fully connected network. All modules incorporate Layer Normalization [42] and Dropout [43] to enhance the training stability.

By jointly mining the time–frequency feature information and utilizing multi-scale convolutional layers with linear operations, the overall design achieves transformer-like modeling capabilities [44]. This approach maintains the detection performance while significantly reducing the computational complexity. The composition and functionality of each module are detailed below.

### 3.2. The Feature Embedding Module

**Figure 3:**
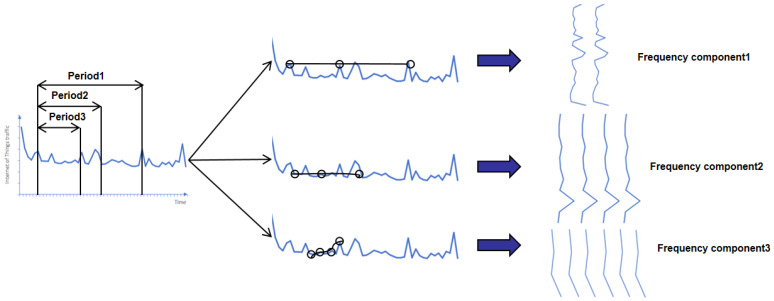
Time–frequency domain transformation graph of network traffic characteristics.

The feature embedding module processes the input features through two parallel pathways: conventional time-domain feature embedding and frequency-domain feature embedding. This dual-path design transforms features such as bursty traffic and periodic scanning into distinctive frequency-domain patterns via separate time–frequency processing. The parallel structure preserves both fine-grained original feature details and global spectral properties. Implementation-wise, the module first applies a sliding-window Fast Fourier Transform (FFT) [45] to the input network traffic sequences, extracting the real component (using a window size of 256 and a step size of 128). This converts the 1D flow characteristics within each window into a frequency-domain energy distribution, generating a 2D time–frequency feature map (Figure 3). Subsequently, linear transformations independently process the time-domain and frequency-domain features. A fully connected layer projects these time–frequency features into a high-dimensional space, with Layer Normalization (LN) and Dropout applied after each transformation to enhance the training stability and mitigate overfitting. Finally, the processed time-domain and frequency-domain features are fused to enrich the model’s multi-dimensional data understanding.

### 3.3. The Depthwise Separable Convolution Block

**Figure 4:**
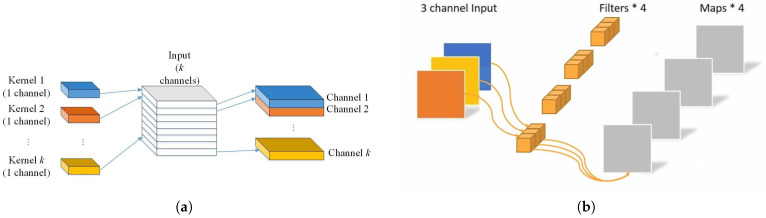
The depthwise separable convolution steps can be divided into channel-by-channel convolution and point-by-point convolution. (**a**) Channel-by-channel convolution, where each convolution kernel corresponds to a channel, respectively, rather than all of the inputs being input into each channel for convolution calculation. (**b**) Point-by-point convolution involves weighted combination of the output feature maps obtained from channel-by-channel convolution in the depth direction to form new feature maps. There will be as many output feature maps as there are convolution kernels.

The module employs depthwise separable convolution for efficient local feature extraction. This technique decomposes standard convolution into depthwise (channel-wise) and pointwise (1 × 1) convolution, capturing multi-granular information while significantly reducing the computational complexity and parameter count without compromising the effectiveness of feature extraction or the detection accuracy. Operationally, the input tensor (batch size × input channels × height × width) first undergoes depthwise convolution using appropriate padding to maintain the spatial dimensions. Here, each input channel is convolved independently with its own kernel, yielding channel-wise features stacked along the channel dimensions. These features then undergo pointwise convolution via 1 × 1 kernels to achieve cross-channel fusion and weighted integration (Figure 4). To ensure training stability, convolution weights are initialized using Kaiming Normal initialization [46] (suitable for GELU activation), while the biases are set to zero.

### 3.4. Basic Blocks

Each basic block employs two depthwise separable convolution blocks, GELU activation functions, and regularization components, all interconnected via residual connections. Within the block, the input data first undergoes Layer Normalization (LN). Subsequently, the normalized features pass through the first depthwise separable convolution block, followed by a GELU activation function and Dropout. This processed output then enters the second depthwise separable convolution block. Finally, the output of this second block merges with the original block input through a residual connection. This design enhances the information fusion across deep layers and the training stability, effectively mitigating gradient vanishing issues in deep networks while preserving critical feature information from the network traffic.

### 3.5. Calculation of the Model Calculations and Parameter Quantities

#### 3.5.1. The Feature Embedding Module Complexity Calculation

The computational complexity of this module lies mainly in the FFT operations and the fully connected layers of LN plus Dropout computed in parallel from the time–frequency domain. LN is shown in Equation (1):

(1) $$y=\frac{x-\mu }{\sqrt{Var(x)+\varepsilon }}*\gamma +\beta$$

where μ stands for the mean, and the scaling γ and offset β are learnable parameters. LN needs addition, subtraction, multiplication, and division computations for the mean, variance, and two parameters. Assuming that the input feature dimensions are d, we can obtain the amount of computation and the parametric quantities as in Equations (2) and (3):

(2) $$FLOPs_{LN}=5*d$$

(3) $$Params_{LN}=2*d$$

The Dropout layer discards neurons only through random masks, so there is no parameter learning. The computational effort comes mainly from the random masking and element-by-element multiplication generated by the training, assuming the size of the input tensor is n. The computational effort is as in Equation (4):

(4) $$FLOPs_{Dropout}=2*n$$

The number of parameters in the fully connected layer is determined by the weight matrix and the bias vector. The computational effort comes mainly from matrix multiplication and bias addition. We initialize all of the weight matrices with a Kaiming normal distribution, and then the bias vector = 0, assuming the size of the input vector is N and the size of the output vector is M, so the computational and parametric quantities of the fully connected layer are expressed in Equations (5) and (6):

(5) $$FLOPs_{FC}=2*N*M$$

(6) $$Params_{FC}=N*M+M=M*\left(N+1\right)$$

#### 3.5.2. Depthwise Separable Convolution Block Complexity Calculation

Traditional standard single convolution is computed as in Equation (7) and the number of parameters is calculated in Equation (8):

(7) $$FLOPs_{Convolution}=D_{K}*D_{K}*M*N*D_{F}*D_{F}$$

(8) $$Params_{Convolution}=M*D_{K}*D_{K}*N$$

Here, $D_{F}$ it is the length of the input features, $D_{K}$ the length of a single convolution kernel, and M represents the number of input channels, while N denotes the number of output channels. Our model uses depthwise separable convolution (DSC), which splits the standard convolution into channel-by-channel and point-by-point convolution, with the computational and parametric quantities as in Equations (9) and (10):

(9) $$FLOPs_{DSC}=FLOPs_{DW}+FLOPs_{PW}=D_{K}*D_{K}*M*D_{F}*D_{F}+M*N*D_{F}*D_{F}$$

(10) $$Params_{DSC}=Params_{DW}+Params_{PW}=M*D_{K}*D_{K}+M*N$$

From the above, Equations (7)–(10), the ratio of the computational complexity of depthwise separable convolution and standard convolution can be obtained using Equation (11):

(11) $$\frac{FLOPs_{DSC}}{FLOPs_{Convolution}}=\frac{Params_{DSC}}{Params_{Convolution}}=\frac{1}{N}+\frac{1}{D_{K}^{2}}$$

#### 3.5.3. An Analysis of the Model’s Complexity

The basic block of our model consists of two depthwise separable convolution blocks and components such as LN, Dropout, and the residual network. The residual network uses element-by-element addition, which is computationally small and nearly negligible. Although the stacking of the model using multiple basic blocks generates some computational growth, experiments show that this has a key role in the improvement in the detection accuracy, and the computational and parameter counts are still significantly reduced compared to those of some models that introduce multiple attention mechanisms. Overall, our model maintains a strong performance in its detection accuracy while minimizing the complexity and is more suitable for lightweight deployment scenarios than other network intrusion detection models.

## 4. Experiments

### 4.1. The Datasets

The NSL-KDD dataset is an improved version of the KDD99 dataset and is widely recognized as a benchmark dataset in intrusion detection research. The dataset contains four main attack categories: probing (Probe), user-to-root (U2R), remote-to-local (R2L), and denial of service (DoS). Specifically, DoS attacks (e.g., Syn Flood) aim to disrupt the availability of a target service; probing attacks (e.g., Port Scanning) focus on gathering information about the network for subsequent attacks; U2R attacks enable privilege escalation by exploiting techniques such as buffer overflow; and R2L attacks (e.g., Password Guessing) attempt unauthorized remote access.The NSL-KDD dataset comprises four subsets consisting of a complete training set containing 125,973 records and a test set containing 22,544 records. Each record contains 43 features, including 41 traffic features (e.g., duration, protocol type, and flag pattern). The label distribution shows a significant category imbalance, where DoS attacks account for 36.46% of the training samples, while U2R and R2L attacks together account for less than 1%. The last two attributes indicate the attack labels and the severity levels, respectively. The number and proportion of categories in the dataset are detailed in Table 2.

The CIC-IDS2018 dataset is a modern intrusion detection dataset constructed under the supervision of the Canadian Institute for Cyber Security (CIC). This dataset was collected over five consecutive days, capturing network traffic data by deploying various network attacks alongside normal traffic in a controlled experimental environment, with a total of 16,232,943 records, of which 13,484,708, or 83.07%, are normal traffic records. The dataset is collected from real experimental scenarios, and each piece of data contains detailed network traffic features, such as the packet size, protocol type, and source and destination IP addresses, and there are 15 different attack types in the dataset, and 71 network traffic features are extracted. In this study, we adopted a stratified random sampling method, allocating 10% of the dataset as the training set, 2% as the validation set, and the remaining 2% as the test set. The number and proportion of categories in the dataset are detailed in Table 3.

### 4.2. Data Processing

For the NSL-KDD dataset, in the data preprocessing phase, the missing values and outliers in the dataset are first thoroughly checked. Subsequently, data cleaning is completed by removing outliers and filling or processing missing values. Given that the dataset contains both continuous and discrete features, for discrete features (e.g., protocol types), the one-hot encoding method is used to convert them into multi-dimensional binary vector forms, while for continuous numerical features, the Min–Max normalization technique is used to scale them to the [0, 1] interval, thus avoiding disproportionate impacts of features with different magnitudes on the model training.

For the CIC-IDS2018 dataset, the data preprocessing process consisted of digitising timestamped features to generate numeric-type features suitable for modeling and removing meaningless traffic identification columns that could trigger model overfitting. In addition, the classification features were coded and the numerical-type features were normalized to a uniform scale to reduce the impact of inter-feature magnitude differences on the model’s performance. The role of different feature scales on the training effect is evaluated further during the model training process.

### 4.3. The Selection of Mixed Sampling Methods

In order to solve the data imbalance problem, traditional oversampling methods have some limitations. The Random Oversampling (ROS) method is prone to model overfitting when dealing with data imbalances, as a few class samples are selected and replicated through random selection. The SMOTE method, on the other hand, generates synthetic samples through linear interpolation, which can prevent overfitting to a certain extent, but it may also trigger further imbalances in the data distribution within the classes and even amplify the noise, thus affecting the classification performance of the model. For this reason, this paper adopts a synergistic optimization strategy that incorporates adaptive synthetic sampling (ADASYN) and boundary cleaning techniques (TomekLinks).

In the oversampling stage, ADASYN dynamically adjusts the synthesis strategy according to the distribution density of various types of samples, identifies sample regions with a higher classification difficulty using a k-nearest neighbor analysis, and generates synthetic samples based on the Gaussian distribution function, in line with the trend in the spatial distribution of the features of the real data, which is particularly strengthened by complementing sparse samples near the decision boundary, effectively avoiding the overfitting problem due to sample repetitions and, at the same time, improving the recognition ability of fuzzy regions.

In the undersampling phase, the TomekLinks mechanism optimizes the inter-class boundaries of the mixed dataset. By calculating the Euclidean distance between pairs of heterogeneous samples, interfering samples that form “pseudo-nearest neighbors” with a few samples in the majority class are accurately located and removed, thus effectively eliminating the noisy data in the area of overlapping classes while preserving the representative features of the samples in the majority class.

**Figure 5:**
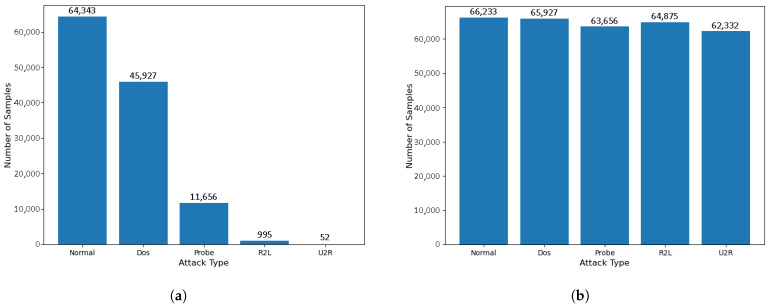
A comparison of the number of categories to the NSL-KDD dataset before and after ADASYN+TomekLinks sampling: (**a**) before ADASYN+TomekLinks sampling and (**b**) after ADASYN+TomekLinks sampling.

**Figure 6:**
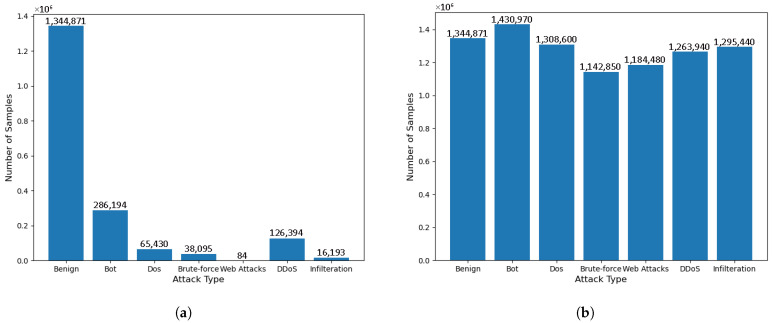
A comparison of the number of categories in the CIC-IDS2018 dataset before and after ADASYN+TomekLinks sampling: (**a**) before ADASYN+TomekLinks sampling and (**b**) after ADASYN+TomekLinks sampling.

This two-stage process of synthesis followed by elimination not only mitigates the quantitative imbalance between classes through adaptive sample generation but also optimizes the structural distribution of the feature space and enhances the representation of a few classes through boundary cleaning, as well as improving the clarity of the classification boundaries, which systematically enhances the model’s efficiency in capturing complex data patterns and its performance in generalizing to them. The sampling results are shown in Figure 5, and the sampling results of pre-labeled classification for the CIC-IDS2018 dataset are shown in Figure 6. As shown in Figure 5b, under the multi-classification task, ADASYN+TomekLinks increased the U2R samples in NSL-KDD from 0.08% normal samples to 93% and the R2L samples from 15.4% normal samples to 97%, alleviating the category imbalance. Figure 6b shows that the Web Attacks samples in CIC-IDS2018 increased from less than 0.01% of the Bengin samples to 88%, while eliminating the overlapping noise between classes.

### 4.4. Selection of the Experimental Hyperparameters and Assessment Indicators

The model training parameters in this study were set as follows: the model training process was carried out using a cross-entropy loss function and the Adam optimizer. The training process employed an early stop strategy and terminated when the validation set loss did not decline for five consecutive rounds. For the NSL-KDD dataset, we set the total number of rounds (epoch) of training to 50 and the batch size to 256. The initial learning rate was set to 0.001, and the learning rate was dynamically adjusted using the cosine annealing strategy, decaying by 50% every five cycles so that the optimizer could converge more accurately. For the CIC-IDS2018 dataset, which had a larger data size, in order to ensure the robustness of the model, we set the total number of rounds (epoch) of practice to 500 and the batch size to 1000 and still adopted an initial learning rate of 0.001 and applied the cosine annealing strategy. We conducted five repeated experiments, and the results were taken as the mean ± standard deviation of the experiments. The evaluation indicators for this study were calculated from the confusion matrix in Table 4.

Where TP = the number of samples where both the predicted and true values are attacks; FN = the number of samples where the predicted value is normal but the true value is an attack; FP = the number of samples where the predicted value predicts an attack but its true value is normal; and TN = the number of samples where both the predicted and true values are normal. The accuracy, precision, recall, and F1 score are used to determine the classification effectiveness of the model. Equations (12)–(15) are calculated as follows:

(12) $$Acc=\frac{TP+TN}{TP+TN+FP+FN}*100\%$$

(13) $$PRE=\frac{TP}{TP+FP}*100\%$$

(14) $$Recall=\frac{TP}{TP+FN}*100\%$$

(15) $$F1=\frac{2*PRE*Recall}{PRE+Recall}*100\%$$

In addition, in order to comprehensively evaluate the model’s attack recognition ability and defence performance in network security scenarios, we judged the model’s performance in real threat scenarios by using the AUC-ROC curve and the AUC-PR curve. The AUC-ROC curve, by calculating the trade-off relationship between the True Positive Rate (TPR) and the False Positive Rate (FPR), is able to reflect the model’s global ability to distinguish between normal and abnormal behaviors under different thresholds. The larger the area under the curve (AUC), the better the overall performance of the model when taking into account multiple types of samples. The AUC-PR curve, on the other hand, focuses on the model’s ability to detect scarce positive samples by depicting the dynamic equilibrium between precision and recall, and its area can be regarded as the average performance in precision under different recall thresholds, which is especially suitable for scenarios with extreme imbalances between positive and negative samples in network security. The two metrics complement each other: the AUC-ROC reveals the stability of the model under the defender’s perspective, while the AUC-PR highlights the actual efficacy in attack detection, and together, they build a multi-dimensional evaluation system for model performance.

### 4.5. A Visualization Analysis of Time–Frequency Characteristics

**Figure 7:**
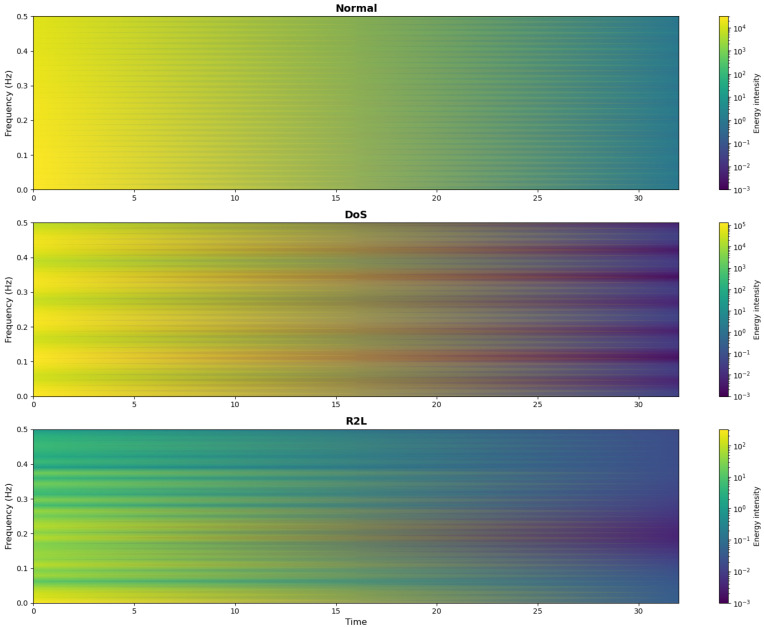
A time–frequency heatmap comparison of the attack types in the NSL-KDD dataset.

To verify the effectiveness of the frequency-domain analysis in intrusion detection, we used the Fast Fourier Transform to generate a time–frequency heatmap comparison between normal traffic and two typical types of attacks (burst DoS attacks and covert R2L attacks). Figure 7 shows the results of the analysis of representative samples in the NSL-KDD dataset. The normal network flow shows stable frequency-domain characteristics, and the changes in the time–frequency heatmap are relatively gentle, without obvious abnormal fluctuations. DoS attacks exhibit distinct high-frequency characteristics in the time–frequency domain. A frequency-domain analysis can effectively capture such instantaneous but high-intensity abnormal patterns. R2L attacks exhibit significant low-frequency characteristics. Such low-frequency anomalies are easily masked by regular traffic in the time domain but can be clearly identified through a frequency-domain analysis. The above visualization results verify the rationality of the time–frequency dual-domain analysis adopted by FFT-RDNet: the high-frequency features of burst attacks and the low-frequency features of covert attacks are highly distinguishable in the frequency domain, while the traditional time-domain models find it difficult to effectively capture these patterns.

### 4.6. Experimental Results

**Figure 8:**
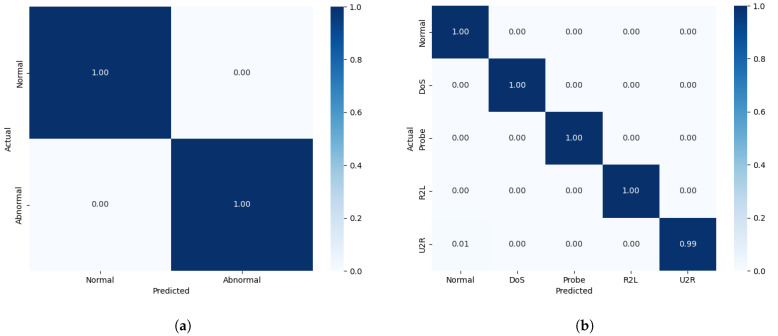
FFT-RDNet binary and multi-classification confusion matrices for the NSL-KDD dataset: (**a**) binary confusion matrix on the NSL-KDD dataset; (**b**) multi-classification confusion matrix on the NSL-KDD dataset.

Binary and multi-classification experiments were performed on the NSL-KDD dataset, which has 41 features and 23 subcategories of attack types, which we consolidated into four different attack types—DoS, R2L, U2R, and probing—and recorded the experimental hyperparameters in the previous section. We compared the model performance and its confusion matrix with those for several other models that have performed well in recent years in binary and multi-classification tasks, as shown in Table 5 and Figure 8, and the experiments demonstrate that the model proposed in this paper outperforms all of the other models across the four evaluation metrics. In addition, we also detected the four types of attacks in the dataset, respectively, and the results are shown in Table 6. It can be seen from the confusion matrix and Table 6 that compared with common attack types such as DoS attacks, low-frequency covert attacks (R2L/U2R) are more difficult to identify, while the detection performance of our model is higher. The reason is that FFT-RDNet takes into account the feature dimensions of the frequency domain and has better recognition capabilities for both high-frequency and low-frequency attacks.

**Figure 9:**
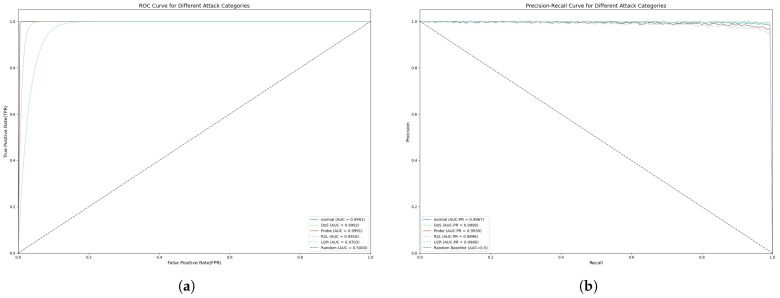
FFT-RDNet’s multi-classification AUC-ROC and AUC-PR on the NSL-KDD dataset: (**a**) AUC-ROC score; (**b**) AUC-PR score.

In order to evaluate the performance of the model in multi-classification intrusion detection, we evaluated the five types of attacks in NSL-KDD using the AUC-ROC curve and the AUC-PR curve, as shown in Figure 9; we found that the model obtains a better performance in most of the categories. The model is more advantageous for identifying more stealthy types of attacks compared to several other methods.

**Figure 10:**
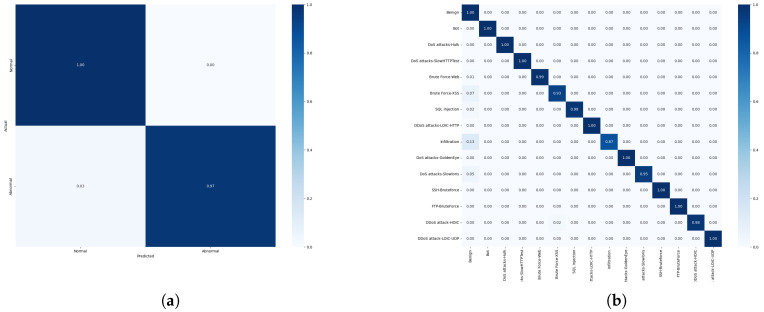
FFT-RDNet binary and multi-classification confusion matrices for the CIC-IDS2018 dataset: (**a**) binary confusion matrix for the CIC-IDS2018 dataset; (**b**) multi-classification confusion matrix for the CIC-IDS2018 dataset.

**Figure 11:**
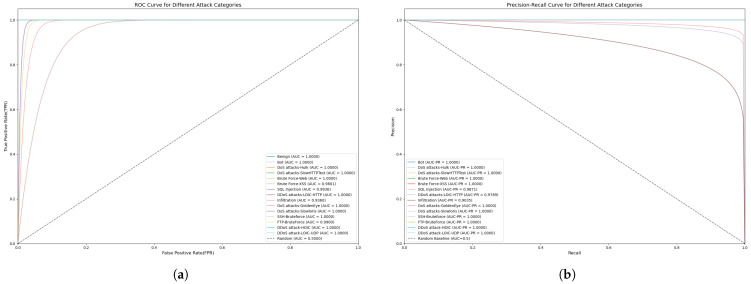
FFT-RDNet’s multi-classification AUC-ROC and AUC-PR for the CIC-IDS2018 datasets: (**a**) AUC-ROC score; (**b**) AUC-PR score.

For the CIC-IDS2018 dataset, considering the large size of this dataset, we performed proportional sampling for each category in each file and used mixed sampling with ADASYN+TomekLinks, which resulted in a new dataset that contained strips of training set data and strips of test and data, containing 71 features and 15 categories. The model performance and confusion matrices for both binary and multi-classification tasks are presented in Table 7 and Figure 10, where our model outperforms all of the other models across all four evaluation metrics. For this dataset, we conducted tests on six types of attacks. The results in terms of the F1 scores are shown in Table 8. For the bot and DoS attack categories, the current model can identify them quite well. We also evaluated the AUC-ROC and AUC-PR metrics for 15 attack types to ensure that the model had a more stable detection performance in real threat scenarios, as shown in Figure 11 specifically. For more covert web attacks and infiltration, the performance of FFT-RDNet and several current mainstream models still has room for improvement.

### 4.7. Ablation Experiments

To verify the effectiveness of each module in our proposed model, we conduct ablation experiments on the NSL-KDD dataset, using the Transformer+CNN+LSTM model proposed above as the baseline. “Ours” refers to the version with all modules intact. We still use the Adam optimizer and train 50 epochs with the learning rate varying with the training process to compare the training efficiency and evaluation metrics of the different versions: (1) Fast Fourier Transform (FFT)+depthwise separable convolution, (2) depthwise separable convolution+ResNet, and (3) FFT+CNN+ResNet. The results are shown in Table 9. It can be seen that each part contributes to the final model’s performance. Each part of FFT-RDNet contributes to the training effect. FFT acquires the frequency-domain information on the features to help the model to extract the feature information from multiple dimensions. Convolutional stacking is used to extract the features from the local to the global level. Residual connectivity improves the running efficiency of the structure and alleviates the problem of gradient vanishing in deep neural networks. We also compared the different quantities of basic blocks, and the results are shown in Table 10. The six-layer structure performs best in terms of accuracy, and the number of model parameters increases significantly when stacking basic blocks. The six-layer structure achieves the optimal balance between accuracy and complexity.

### 4.8. Edge Deployment Feasibility Validation

To address the practical deployment requirements for resource-constrained IoT devices, we evaluated rigorous latency and memory benchmarks using the industry-standard edge simulation tools. The FFT-RDNet model was converted into TensorFlow Lite format with post-training quantization (FP16 precision) and deployed on a Raspberry Pi 4B (Broadcom BCM2711, 4 GB RAM) running Raspberry Pi OS Lite. We measured the average inference latency over 500 iterations using synthetic inputs matching the NSL-KDD feature dimensions. Comparative tests included ViT and CNN-BiLSTM as computationally intensive baselines. The results in Table 11 demonstrate our model’s hardware efficiency.

## 5. Conclusions and Future Work

This study proposes a network attack detection method based on a time–frequency domain analysis, which is not only applicable to the dataset and the experimental environment applied in the experiments but also demonstrates a strong generalization performance in real network environments and attack scenarios. The ADASYN-Tomek Links hybrid sampling strategy shows particular effectiveness in balancing a dataset dominated by regular operational data and successfully mitigates the implicit endpoint leakage of false negatives while maintaining the temporal integrity of critical infrastructure communications. The characteristics of the network traffic are analyzed in the dual dimensions of the time–frequency domains, combined with stepwise extraction of the multi-scale features from the local to the global level. The model structure is designed to be flexible, adapting to network changes across different scenarios, including cloud computing services and IoT environments.

However, FFT-RDNet is still limited by the need to preset the FFT windows, and there are still problems when facing complex traffic environments in different scenarios. We also acknowledge that the reliance on established datasets like NSL-KDD and CICIDS2018, despite their widespread use for benchmarking, introduces potential constraints; these include outdated attack types in NSL-KDD and known feature redundancies, which may not fully represent the complexity and evolution of contemporary network threats. In addition, on ultra-low-power consumption devices (such as MCUs < 100 MHz), this model still needs to undergo further quantization compression. In the future, we will focus on expanding FFT-RDNet, researching adaptive segmented FFT windows, and conducting evaluations on more diverse and representative datasets encompassing emerging threat landscapes to validate its generalization further. We will also pursue further quantification and compression of the models to support real-time intrusion detection in dynamic Internet of Things environments and deployment on ultra-low-power devices. We are porting FFT-RDNet to ESP32 using CMSIS-DSP and will publish real-device benchmarks in subsequent work. Finally, we aim to develop an end-to-end framework that integrates preprocessing and feature learning, thereby enabling joint optimization to improve the data quality and the model’s overall robustness.

## Figures and Tables

**Table 1:** Comparison of existing network intrusion detection model methods.

Year	Methods	Datasets	Balancing Methods	Feature Dimension	Parameter Quantity	Limitations
2017	CNN-based	KDDcup99, NSL-KDD	–	1D	500 K–1 M	Limited sequential modeling, poor handling of temporal dynamics
2019	FFT-based	NSL-KDD	–	1D (only frequency domain)	200–300 K	Limited to the frequency domain
2022	BiLSTM	NSL-KDD	–	1D (only time domain)	1–2 M	Limited to the time domain, limited to NSL-KDD
2023	CBF-IDS [40]	UNSW-NB15 CIC-IDS2017 NSL-KDD	Focal loss	2D	1.8–2.2 M	Long training time Poor interpretability
2024	IDS-INT	UNSW-NB15 CIC-IDS2017 NSL-KDD	SMOTE	1D	3–5 M	High computational cost, large data requirements, overfitting risk
2024	CNN-BiLSTM	UNSW-NB15	Weighted loss	2D (time and space dimensions)	1.8–2.2 M	Sensitive to long sequences
2024	ViT models	NSL-KDD	Bayesian optimization	2D (two-dimensional images)	50–80 M	Long training time
2025	ours	NSL-KDD CIC-IDS2018	ADASYN and Tomeklinkes	2D (time–frequency domain)	350–500 K	The size of the FFT window needs to be preset

**Table 2:** NSL-KDD dataset composition.

Dataset	Quantity and Proportion	Normal	DoS	Probe	R2L	U2R
KDDTrain+	Number scale	67,343 53.46%	45,927 36.46%	11,656 9.25%	995 0.79%	52 0.04%
KDDTest+	Number scale	9711 43.08%	7458 33.08%	2421 11.77%	2654 0.89%	200 0.89%

**Table 3:** CIC-IDS2018 dataset composition.

Category	Total Size	Total Rate	Train Size	Test Size
Benign	13,448,708	83.07%	1,344,871	267,762
Bot	286,191	1.76%	286,194	5705
DoS attacks-Hulk	461,912	2.85%	46,191	9205
DoS attacks-SlowHTTPTest	139,890	0.86%	13,989	2795
Brute Force-Web	611	0.004%	61	13
Brute Force-XSS	230	0.001%	23	6
SQL Injection	87	0.001%	9	2
DDoS attacks-LOIC-HTTP	576,192	3.55%	57,619	11,578
Infiltration	161,934	1%	16,193	3190
DoS attacks-GoldenEye	41,508	0.26%	4151	827
DoS attacks-Slowloris	10,990	0.07%	1099	223
SSH-Bruteforce	187,589	1.16%	18,759	3755
FTP-Bruteforce	193,360	1.19%	19,336	3889
DDoS attacks-HOIC	686,023	4.23%	68,602	13,753
DDoS attacks-LOIC-UDP	1730	0.01%	173	38

**Table 4:** Confusion matrix of network intrusion detection.

Confusion Matrix		Predicted Value	
		**Normal**	**Attack**
True value	Normal	TN	FP
	Attack	FN	TP

**Table 5:** A comparison of the multi-classification performance of FFT-RDNet and other existing models on the NSL-KDD dataset.

	Accuracy	Precision	Recall	F1
GMM-WGAN [47]	84.65%±1.07%	85.13%±1.33%	84.65%±1.07%	83.95%±1.28%
XGB [48]	95.54%±1.12%	92.61%±2.37%	95.54%±1.12%	93.41%±0.89%
IDS-INT	98.45%±0.52%	98.00%±0.52%	99.00%±0.52%	98.00%±0.49%
Res-TranBiLSTM [49]	90.99%±2.54%	91.39%±1.12%	90.94%±2.54%	90.89%±2.06%
Transformer-based [50]	97.84%±0.1%	97.95%±0.12%	97.72%±0.1%	97.83%±0.07%
Ours	99.56%±0.06%	99.56%±0.06%	99.56%±0.06%	99.54%±0.06%

**Table 6:** A comparison of the F1 scores of four types of attacks for the NSL-KDD dataset.

	Ours	IDS-INT	Transformer-Based
DoS	99.78%±0.06%	98.53%±1.12%	98.01%±0.07%
Probe	99.63%±0.06%	98.14%±0.09%	98.28%±0.12%
R2L	99.27%±0.06%	96.76%±1.12%	95.26%±0.83%
U2R	98.47%±0.06%	96.13%±0.94%	94.51%±1.14%

**Table 7:** A comparison of the multi-classification performance of FFT-RDNet and other existing models on the CIC-IDS2018 dataset.

	Accuracy	Precision	Recall	F1
CNN+LSTM [51]	97.11%±0.12%	96.83%±0.12%	97.11%±0.12%	97.00%±0.12%
U-net [52]	97.77%±0.07%	97.94%±0.07%	97.53%±0.07%	97.73%±0.07%
HPO+DNN [53]	95.79%±0.18%	95.38%±0.18%	95.79%±0.18%	95.11%±0.18%
Novel CNN [54]	97.20%±0.15%	99.10%±0.04%	97.20%±0.15%	95.30%±0.15%
HDLNIDS [55]	98.90%±0.09%	98.60%±0.11%	99.16%±0.09%	98.83%±0.10%
Ours	99.12%±0.06%	99.06%±0.06%	99.12%±0.06%	98.82%±0.06%

**Table 8:** F1 score comparison for seven types of attacks on the CIC-IDS2018 dataset.

	Ours	HDLNIDS	U-Net
Bot	99.41%±0.06%	99.53%±0.02%	99.01%±0.07%
DDoS	97.05%±0.06%	96.94%±0.06%	97.28%±0.07%
Infiltration	91.24%±0.06%	90.76%±0.09%	90.26%±0.07%
DoS	99.23%±0.06%	99.13%±0.09%	99.11%±0.07%
Web Attacks	96.27%±0.06%	96.76%±0.04%	96.26%±0.07%
Brute Force	97.89%±0.06%	97.96%±0.05%	96.26%±0.07%

**Table 9:** Performance of different modules of FFT-RDNet on NSL-KDD dataset.

	Accuracy	Precision	Recall	F1
IDS-INT	98.45%±0.52%	98.00%±0.52%	99.00%±0.52%	98.00%±0.49%
FFT+depthwise separable convolution	99.22%±0.22%	99.32%±0.26%	99.22%±0.22%	99.27%±0.25%
Depthwise separable convolution+ResNet	98.36%±0.12%	98.17%±0.08%	98.36%±0.12%	98.04%±0.09%
FFT+CNN+ResNet	99.32%±0.06%	99.42%±0.1%	96.32%±0.06%	99.36%±0.08%
Ours	99.56%±0.06%	99.56%±0.06%	99.56%±0.06%	99.54%±0.06%

**Table 10:** Performance comparison of different numbers of Basic Blocks on NSL-KDD dataset.

Module Combination	Accuracy	Parameter Quantity
Four Basic Blocks	98.41%±0.06%	300 K
Six Basic Blocks	99.56%±0.06%	500 K
Eight Basic Blocks	99.44%±0.06%	800 K

**Table 11:** Edge deployment performance on Raspberry Pi 4B.

Module	Latency (ms)	Peak RAM (MB)	Model Size (MB)
FFT-RDNet	12.3±0.4	45.2±0.6	1.1
ViT	210.5±3.2	312.8±2.1	48.7
CNN-BiLSTM	58.7±1.1	127.4±1.8	8.9

## Data Availability

The NSL-KDD datasets generated for this study can be located at the following URL: https://www.unb.ca/cic/datasets/nsl.html (accessed on 20 February 2025). The CIC-IDS2018 datasets generated for this study can be located at the following URL: https://www.unb.ca/cic/datasets/ids-2018.html (accessed on 25 February 2025).
